# Hernie discale cervicale post traumatique

**DOI:** 10.11604/pamj.2015.22.295.7833

**Published:** 2015-11-24

**Authors:** Rachid Ammor, Assou Ajja

**Affiliations:** 1 Neurosurgery Department, Military Hospital My Ismail, Meknes, Morocco

**Keywords:** Tétraparésie post traumatique, IRM cervicale, hernie discale cervicale

## Image en medicine

Il s'agit d'un patient de 54 ans, tabagique chronique, victime d'un accident de la voie publique avec réception sur le crane. L'examen clinique trouve un patient conscient, avec tétraparésie à prédominance brachiale (grade C de Frankel) et irritation pyramidale (réflexes ostéotendineux vifs au niveau supérieur et inférieur). Le bilan radiologique initial (radios standards et scanner) était sans particularités. Devant ce tableau clinique, une IRM cervicale a été réalisée et a objectivé une hernie cervicale compressive en C5-C6 avec contusion centromédullaire (A). Le patient a été opéré par voie antérieure avec réalisation d'une discectomie C5-C6 et mise en place d'un greffon iliaque et une plaque cervicale (B). L'évolution postopératoire était favorable ; la déambulation a été possible quatre jours après l'opération. L'hernie discale cervicale post-traumatique est rare (3,8% des traumatismes cervicaux). Quand elle est isolée, les investigations paracliniques peuvent passer à coté de cette lésion et c'est l'IRM qui permet de poser le diagnostic et d'apprécier le degré de compression médullaire et radiculaire.

**Figure 1:**
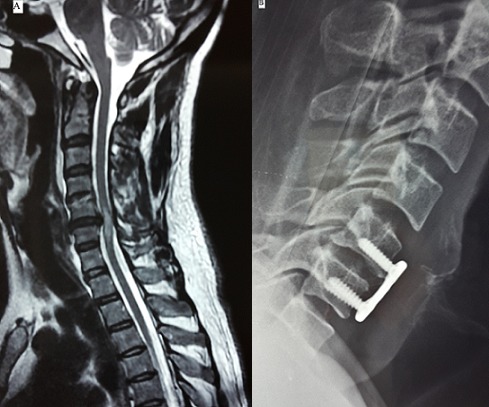
(A) IRM cervicale coupe sagittale en séquence pondérée T2 montrant une hernie discale en C5-C6 comprimant la moelle qui est siège d'une contusion centromédullaire; (B) radiographie cervicale de profil en postopératoire montrant la plaque cervicale et le greffon en C5-C6

